# Psychiatric Disorder in a Patient With Beckwith-Wiedemann Syndrome: A Case Report

**DOI:** 10.7759/cureus.40377

**Published:** 2023-06-13

**Authors:** Deepam Kundal, Luba Leontieva, James L Megna

**Affiliations:** 1 Psychiatry, State University of New York Upstate Medical University, Syracuse, USA; 2 Psychiatry, State University of New York Upstate Medical University, Syracuse, USA; 3 Psychiatry and Behavioral Sciences, State University of New York Upstate Medical University, Syracuse, USA

**Keywords:** stress-related mental disorders, pheochromocytoma, cannabis induced psychosis, major depressive disorder (mdd), borderline personality disorder, beckwith-wiedemann syndrome

## Abstract

Patients with Beckwith-Wiedemann syndrome (BWS) often suffer from pheochromocytoma and hypoglycaemia and are vulnerable to disorders associated with the hypothalamic-pituitary-adrenal axis (HPA), such as major depressive disorder, generalised anxiety disorder, borderline personality disorder, etc. Features of pheochromocytoma even overlap with features of anxiety disorders, panic disorders, etc. These patients undergo multiple major surgeries under general anaesthesia at a very young age due to recurrent tumours that can affect their behavioural and emotional development. Depriving them of much-needed medical and emotional support negatively impacts their physical and psychological well-being.

In this case report, we present the case of a 23-year-old woman with Beckwith-Wiedemann syndrome (BWS) who underwent major surgeries such as partial pancreatectomy, adrenalectomy, osteotomy, and paraganglioma resection at an early age. She was neglected by her parents and spent her childhood in an abusive environment. All these factors could have increased her vulnerability to mental health problems. She was diagnosed with borderline personality disorder, major depressive disorder, unspecified trauma, stressor-related disorders, cannabis use disorder, and cannabis-induced psychotic symptoms. This report emphasises the role of medical comorbidity in a patient presenting with borderline personality disorder.

## Introduction

Beckwith-Wiedemann Syndrome (BWS) is a genetic disorder caused by dysregulated gene transcription in two imprinted domains on chromosome 11p15.5 [1]. It was named after two clinicians: John Bruce Beckwith (an American pathologist) and Hans-Rudolf Wiedemann (a German paediatrician), who reported the condition independently in 1963 and 1964, respectively [2]. It is a rare condition with an estimated prevalence of one in 10,000 [3].

The features of BWS are divided into major and minor findings. Some of the common major findings include macrosomia, macroglossia, hemi-hyperplasia, omphalocele, embryonal tumour, visceromegaly involving one or more intra-abdominal organs including the liver, spleen, kidneys, adrenal glands, and/or pancreas, cytomegaly of the foetal adrenal cortex, etc. Findings considered minor are prematurity, neonatal hypoglycemia, vascular lesions including nevus simplex or haemangiomas, characteristic facies including midface retrusion and infraorbital creases, etc. [1].

Various attempts have been made to define BWS, but no consensus exists for clinical diagnostic criteria [4,5]. The most acceptable definition states that to be diagnosed with BWS, one should either fulfil "three major or two major plus one minor criterion" or "an epigenetic or genomic alteration leading to an abnormal methylation at 11p15.5 or a heterozygous BWS-causing pathogenic variant in CDKN1C in the presence of one or more clinical findings" [1].

Solid tumors such as pheochromocytomas, are significantly associated with an increased risk of depression and anxiety disorders. A dysfunctional noradrenergic (catecholamine) system is hypothesised to be the common factor between pheochromocytoma and depressive and anxiety disorders [6]. Undergoing major surgeries at a young age has also been significantly associated with post-traumatic stress disorder (PTSD), depression, and anxiety symptoms [7].

Here we report a case of a young female patient with Beckwith-Wiedemann syndrome (BWS) with a history of multiple surgeries such as partial pancreatectomy, adrenalectomy, shortening osteotomy, and paraganglioma resection with lymph node dissection during childhood and adolescence, in the setting of various psychiatric diagnoses such as borderline personality disorder (BPD), major depressive disorder (MDD), cannabis-induced psychotic disorder, and unspecified trauma and stressor-related disorder. As per the authors’ knowledge, there are no previous studies published discussing psychiatric comorbidities in a patient with BWS.

## Case presentation

The patient is a 23-year-old Caucasian female with reported psychiatric diagnoses of borderline personality disorder (BPD), post-traumatic stress disorder (PTSD), adjustment disorder, cannabis use disorder, generalised anxiety disorder (GAD), major depressive disorder (MDD), and multiple suicide attempts. She voluntarily admitted herself to an acute psychiatry unit with complaints of sadness, crying spells, lack of energy, decreased sleep and appetite, panic attacks, suicidal ideation, and worsening command hallucinations over the past four months. The patient had a car accident four months ago and fractured her right talus bone. Due to this, she was unable to work, which led to financial problems and housing insecurity. She also broke up with her boyfriend around the same time, after 10 years of a relationship.

Due to these stressors, she started feeling depressed and anxious approximately four months ago, and it gradually kept on increasing. The patient felt angry and emotionally unstable all the time. Her sleep and appetite decreased significantly. She felt like doing nothing all day and kept lying in bed. Her auditory and visual hallucinations, which started approximately five to six years ago, worsened during this time, which might be associated with her increased use of cannabis. The patient has been using cannabis every day for the past eight to nine years and would increase the amount during periods of depression and anxiety.

The patient had not seen an outpatient psychiatrist and had not taken any psychiatric medication since 2018, after her last psychiatric hospitalisation. During this time, the patient reported having sadness and anxiety symptoms but continued her job and kept using cannabis to calm herself.

On mental status examination, the patient was a 23-year-old thinly built Caucasian woman who was well-kempt and cooperative. Her speech was spontaneous, goal-directed, and fluent. The patient reported her mood to be "sad, anxious, and felt like crying". Her affect was depressed, with a restricted range, and congruent with her mood. The patient did not have any abnormalities in her thought process. She reported feelings of hopelessness and helplessness, along with suicidal ideation without any plan. She wanted to get help before she could harm herself. She reported having auditory and visual hallucinations daily for the past three to four months, which had been intermittently occurring previously. She would often hear "a raspy voice of an old guy calling her name and telling her to kill herself" along with "faint, unintelligible whispers". Sometimes, she would also see "black figures without faces," which she felt were following her. The patient had good cognition. Her insight and judgement were poor.

Family history

The patient’s biological mother had a history of depression and was receiving treatment which was not known to the patient. Her mother and maternal grandmother had histories of suicide attempts. The patient’s biological father had a history of alcohol abuse. 

Past history

She first started exhibiting depressive and anxiety symptoms at the age of 14, when she was diagnosed with pheochromocytoma. She started using marijuana daily, multiple times a day, around the same time. She started having intermittent auditory and visual hallucinations at the age of 17-18, which seem to be associated with cannabis use as there has never been a period of abstinence.

The patient has a history of four psychiatric hospitalisations since 2016, which included two attempted suicides by overdose. She has been prescribed multiple trials of medications such as fluoxetine, citalopram, mirtazapine, venlafaxine, and quetiapine in the past.

She stopped her medications and did not follow up with psychiatric appointments near the end of 2018, as she felt that medications were not helping her and that she would rather use cannabis to cope with anxiety and stress.

Personal and social history

The patient was born preterm by normal vaginal delivery and was diagnosed with BWS at birth. She had a pancreatic tumour removed just after birth. She met her developmental milestones on time. Her parents got divorced when she was five years old, and she and her sister lived with their father and stepmother after being abandoned by their mother. She was physically, verbally, and emotionally abused by her stepmother.

She moved back to her mother’s house at the age of 14. She completed a certified nursing assistant course and has worked in many jobs since the age of 16. She started living in her apartment at the age of 19. She was in a relationship for eight years that she felt was emotionally abusive. About two years ago, she broke up with him and dated another guy for four months who was physically abusive to her. She reconciled with her previous boyfriend. She broke up with him four months ago when she found out that he was cheating on her and stealing money from her.

Medical and surgical history

She started having symptoms such as headaches, blurry vision, hypoglycaemia, dizziness, palpitations, orthostatic hypotension, etc. at the age of 12. She underwent major surgeries such as left and right partial adrenalectomy for pheochromocytoma, shortening osteotomy of the femur for left lower extremity hemihypertrophy, and paraganglioma resection between 2014 and 2015. Her other medical problems included delayed menarche, nocturnal enuresis, and chronic hyperbilirubinemia.

Interventions in the unit

The patient was admitted for nine days to an acute psychiatry unit with complaints of depressive and anxiety symptoms along with auditory and visual hallucinations, which seemed to be induced by cannabis use rather than being a symptom of any primary psychotic disorder as she had been continuously using cannabis for the past nine years in variable amounts.

The patient had a continuous history of recurrent self-harming behaviour, marked impulsivity with affective instability, mood reactivity, and frequent displays of anger since the age of 14, which was corroborated by her mother. Although the patient could not tell the exact occurrence of depressive episodes, there was a significant history of multiple episodes of sadness in the past lasting for four to five months when the patient would feel like crying all the time and did not feel the energy to do anything. Her appetite and sleep would reduce significantly, and she had suicidal ideations. The patient never felt that her sadness was completely resolved; there was a significant period in between episodes during which she felt normal with or without medication. Although the patient reported significant physical and emotional trauma in the past, she did not fulfil the criteria for PTSD. So, a diagnosis of borderline personality disorder, major depressive disorder, cannabis use disorder, cannabis-induced psychotic symptoms, and unspecified trauma and stressor-related disorders was formulated.

The aim of the admission was tailored more towards providing a therapeutic milieu where the patient could temporarily distract herself from ongoing stressors and get time to recollect herself in a reassuring and validating environment. The patient showed regular attendance in dialectical behaviour therapy (DBT) groups and was advised to continue her sessions in outpatient follow-ups. Medications were prescribed for symptomatic management, such as mirtazapine 7.5mg, melatonin 3mg, and hydroxyzine 50mg for sadness, anxiety, and sleep. Aripiprazole 5mg and prazosin 1mg were also added to the treatment plan to manage hallucinations and nightmares, respectively. The patient reported that her hallucinations were completely resolved during her hospital stay, and after psychoeducation, she understood that they were associated with cannabis use. Consequently, she was highly motivated to stop her cannabis use. Once the patient attains a period of sobriety and more stabilisation, a plan to initiate trauma-focused psychotherapy will be made to promote psychological healing. The patient was discharged with the same medications and referred to an outpatient clinic, and it could not be ascertained if she was compliant with her follow-up appointments.

## Discussion

There are various theories postulating the aetiology of borderline personality disorder, such as a lack of resilience to psychological stressors as a result of a combination of genetic vulnerability and a chronically invalidating environment, a lack of integration in early maternal relationships, etc. [8]. Recent studies suggest stress due to early childhood trauma leads to a dysfunctional hypothalamic-pituitary-adrenal (HPA) axis, which seems to be associated with BPD [9]. Endocrine conditions like pheochromocytoma resemble states of acute stress with overlapping features like anxiety, tremors, palpitations, headaches, and dizziness [10]. So, it is understandable that having a biological condition like BWS with pheochromocytoma that mimics a dysregulated HPA axis can make an individual vulnerable to disorders like BPD or depression. Another consequence of having BWS is undergoing major surgeries under general anaesthesia at a very early age, which has proven to result in long-term neurodevelopmental deficits, especially behavioural problems and symptoms of PTSD in some individuals, increasing vulnerability to mental health disorders [11,12]. This patient’s mental health condition might be a result of a combination of all these factors, thus showing how biological factors are intertwined with psychosocial triggers in the development of the psychiatric disorder. Patients with BPD have a higher prevalence of MDD compared to the general population, with an earlier onset of depression that has higher severity and longer duration and is less responsive to antidepressants, along with raised aggression towards themselves, resulting in worse social impairment [13,14]. 

This patient’s mental state and personality development can be understood as a confluence of various factors, such as having BWS, a family history of depression, abusive childhood experiences, and abandonment by the mother at a very early age. The hormonal impact of pheochromocytoma and recurrent hypoglycaemic episodes, in combination with undergoing multiple surgical treatments at a very young age, laid the groundwork for emotional disorders. Being denied care and support on top of that, which was of paramount importance to this individual, intensified her condition, creating a conflict between trust and mistrust, resulting in disturbances in her attachment formation, self-image, and coping mechanisms. All these factors probably help to explain her early presentation of borderline personality disorder, major depression, and cannabis use disorder.

## Conclusions

A condition like BWS becomes a physiological predisposing factor in the development of mental health disorders directly and indirectly by affecting normal hormonal balance and requiring major surgeries in early childhood, respectively. This, combined with factors like childhood traumatic experiences, abuse, and neglect by parents, can lead to maladaptive psychological and emotional development in children. Psycho-educating parents and guardians of children with BWS and making them aware of the mental health problems that may co-occur is the primary aim of this case report. Furthermore, conducting research to screen for mental health issues in patients with BWS would be beneficial to find any association between them, thus advancing our understanding and management of the disorder.
